# Detection of minimal residual disease by next generation sequencing in AL amyloidosis

**DOI:** 10.1038/s41408-021-00511-6

**Published:** 2021-06-21

**Authors:** Shayna Sarosiek, Cindy Varga, Allison Jacob, Maria Teresa Fulciniti, Nikhil Munshi, Vaishali Sanchorawala

**Affiliations:** 1 Section of Hematology & Medical Oncology, Boston Medical Center, Boston, MA USA; 2 Amyloidosis Center, Boston University School of Medicine and Boston Medical Center, Boston, MA USA; 3 The John C Davis Myeloma and Amyloid Program, Tufts Medical Center, Boston, MA USA; 4 Division of Hematology-Oncology, Tufts Medical Center, Boston, MA USA; 5 Adaptive Biotechnologies, Seattle, WA USA; 6 Division of Hematologic Malignancies, Dana-Farber Cancer Institute, Boston, MA USA

**Keywords:** Haematological cancer, Risk factors

Dear Editor,

Although treatment for light chain (AL) amyloidosis targets clonal plasma cells with the goal of achieving a hematologic complete response (CR) and improving organ response, as well as overall survival [1], some patients do not have organ improvement despite a satisfactory hematologic response. This persistence or worsening of organ dysfunction is potentially related to residual, low-level disease. Improved outcomes may be achieved with deeper free light chain responses [2–5]. The optimal goal for a deep hematologic response is unclear, but may include achievement of a difference in free light chains <10 mg/L, an involved free light chain level ≤20 mg/L, or achievement of minimal residual disease (MRD) negativity [2–6].

In multiple myeloma it is known that achieving MRD negativity can improve patient outcomes [7]. This has not yet been validated in AL amyloidosis. Additionally, the optimal mode of MRD testing is unclear. Next generation sequencing (NGS) is a sensitive manner of detecting MRD in multiple myeloma [7] but the utility of NGS in AL amyloidosis, which has a significantly lower tumor burden, remains to be seen. We designed a study to explore the use of NGS in AL amyloidosis.

Forty-five newly diagnosed patients with suspected AL amyloidosis consented for this trial (NCT02716103) between 2016 and 2019. Nine patients were excluded: six without systemic AL amyloidosis, two with concurrent multiple myeloma, and one with prior treatment. An initial feasibility study was conducted. Five milliliters of blood and bone marrow aspirate were collected from ten patients and processed for CD138 selection and DNA isolation/purification. Samples were sent to Adaptive Biotechnologies Inc. (Seattle, WA) for initial clonal identification using the clonoSEQ Assay. Genomic DNA was amplified by implementing consensus primers targeting multiple loci: IGH complete (IGH-VDJH), IGH incomplete (IGH-DJH), immunoglobulin κ (IGK), and immunoglobulin λ (IGL) [8]. The amplified product was sequenced and a clone identified based on frequency [8]. The initial feasibility study was deemed successful based on discovery of a clone in ≥3 of the first ten patients. Twenty-seven additional patients were enrolled and had clonal identification via the same process. Patients with a trackable clone on initial identification sample had specimens sent for MRD testing using the same assay as pretreatment samples with dominant rearrangements quantified per total nucleated cells. Hematologic and organ responses were assessed at time of MRD testing using current response criteria [9, 10].

Clinical characteristics of the 36 eligible patients are shown in Table 1. clonoSEQ identified trackable clones in the blood or bone marrow in 31/36 patients (86%) prior to treatment (Table 1). Four patients had ≥1 trackable sequence in the blood (range, 1–5) and 29 had ≥1 trackable sequence in the marrow (range, 1–7). Of the four patients with clones in the blood, one was not simultaneously detected in the marrow. Of those with no detectable clone, three had no light chain restriction by immunohistochemistry of the bone marrow. No other correlation was noted between the successful detection of a clone and standard measures of disease.

**Table 1:** Baseline patient characteristics.

Patient #	SIFE	SPEP (g/dL)	UIFE	UPEP (mg/day)	dFLC	Plasma cell % on bone marrow biopsy (clonal restriction indicated)	Trackable clone on initial sample?	Peripheral blood clone detected?	Bone marrow clone detected?
1	IgG L	0.5	IgG L	Neg	26.6	5–10% lambda	Yes	No	Yes
2	L	Neg	L	Neg	9942.5	inadequate	Yes	No	Yes
3	L	Neg	L	Neg	1045.3	15–20% lambda	Yes	Yes	Yes
4	IgG L and L	0.3	Neg	Neg	243	20% lambda	Yes	Yes	Yes
5	Neg	Neg	L	7	62.7	20% lambda	Yes	No	Yes
6	IgG L	0.44	L	Neg	51.5	10% lambda	Yes	No	Yes
7	IgA L	Neg	Neg	Neg	1.5	5–10% no predominance	No	No	No
8	IgG L	Neg	IgG L	Neg	33.5	20–25% lambda	Yes	No	Yes
9	L	Neg	L	Neg	80.5	5–10% lambda	No	No	No
10	IgG K	Neg	K	Neg	765.4	15% kappa	Yes	Yes	No
11	IgG L	0.22	IgG L	228	141.3	20–25% lambda	Yes	No	Yes
12	Neg	Neg	L	332	131.4	20% lambda	Yes	No	Yes
13	IgG L	0.54	L	Neg	49	15–20% lambda	Yes	No	Yes
14	IgG L	0.84	Neg	Neg	81	5% lambda	Yes	Yes	No
15	Neg	Neg	Neg	Neg	52	no predominance	Yes	No	Yes
16	IgG L	1.9	Neg	Neg	13.8	10–15% lambda	Yes	No	Yes
17	Neg	Neg	Neg	Neg	480.8	10–15% kappa	Yes	No	Yes
18	IgD L and L	Neg	L	Neg	137.6	30–40% lambda	Yes	No	Yes
19	IgG L	0.26	Neg	Neg	106.4	30–40% lambda	Yes	No	Yes
20	IgG L	0.92	L	59	80.3	30% lambda	Yes	No	Yes
21	IgM K	0.3	Neg	Neg	30.3	5% kappa	Yes	No	Yes
22	IgG L	0.82	Neg	Neg	28.9	5–10% lambda, 25% B cells	Yes	No	Yes
23	IgM L	1.01	L	Neg	7.5	10–15% lambda, 10% B cells	Yes	No	Yes
24	IgG L	1.34	Neg	Neg	5.7	20–25% lambda	Yes	No	Yes
25	L	Neg	L	200	287.6	15–20% lambda	Yes	No	Yes
26	Neg	Neg	Neg	Neg	93.1	10–15% kappa	No	No	No
27	IgG K	1.17	IgG K	260	22.2	30% kappa	Yes	No	Yes
28	Neg	Neg	Neg	Neg	73.3	5–10% no predominance	Yes	No	Yes
29	L	Neg	L	Neg	152.5	25% lambda	Yes	No	Yes
30	L	Neg	L	72	2203.1	30–40% lambda	Yes	No	Yes
31	IgA L and L	0.1	IgA L and L	Neg	153.9	5–10% no predominance	No	No	No
32	IgA K	0.63	IgA K	Neg	82.4	25% kappa	Yes	No	Yes
33	L	Neg	L	99.8	346.2	30% lambda	Yes	No	Yes
34	Neg	Neg	L	Neg	49.2	5% no predominance	No	No	No
35	L	Neg	L	2169	286.4	10–15% lambda	Yes	No	Yes
36	IgG K and L	Neg	Neg	Neg	236.1	10–15% lambda	Yes	No	Yes

*SIFE* Serum immunofixation electrophoresis, *SPEP* serum protein electrophoresis, *UIFE* urine immunofixation electrophoresis, *UPEP* urine protein electrophoresis, *dFLC* difference in involved to uninvolved serum free light chain, *neg* no monoclonal protein detected, *L* lambda, *K* kappa.

Of the patients with an identifiable clone prior to treatment, eight passed away and ten did not return for follow-up. The remaining thirteen patients had posttreatment testing. Follow-up specimens were obtained at a median of 447 days (range, 147–918) from initial testing. Hematologic response at follow-up was as follows: four hematologic CR, eight very good partial response (VGPR), and one partial response (Table 2). Of the 12 patients with hematologic CR or VGPR, 11 had MRD positivity. Three patients (25%) had ≥1 trackable peripheral blood clone (range, 1–5) and 11 patients (92%) had ≥1 bone marrow clone (range, 1–7). One patient initially had only a trackable clone in the blood but was found to have the same clone in the blood and marrow posttreatment. The one patient with MRD negativity had attained a VGPR.

**Table 2:** MRD testing status.

Patient #	Hematologic status at follow-up (abnormal hematologic parameters listed)	Number of days between identification specimen and MRD test	Trackable clone on follow-up sample?	PB clone detected at follow-up?	BM clone detected at follow-up?	Renal response?	Cardiac response?
3	VGPR (+SIFE)	623	Yes	Yes	Yes	N/a	N/a
4	VGPR (+SIFE, 5% lambda plasma cells in marrow)	770	Yes	Yes	Yes	Yes	Yes
5	VGPR (+UIFE)	608	No	No	No	No	N/a
8	CR	686	Yes	No	Yes	Yes	No
10	VGPR (+SIFE)	918	Yes	Yes	Yes	Yes	Yes
11	VGPR (+SIFE, 5–10% lambda plasma cells in marrow)	532	Yes	Yes	Yes	Yes	N/a
12	CR	238	Yes	No	Yes	Yes	N/a
13	VGPR (+SIFE, +UIFE, 15% lambda plasma cells in marrow)	238	Yes	No	Yes	No	N/a
16	VGPR (+SIFE)	357	Yes	No	Yes	N/a	Na
18	CR	147	Yes	No	Yes	No	N/a
25	PR (+SIFE, +UIFE, 5% lambda plasma cells in marrow)	447	Yes	Yes	Yes	Yes	No
27	VGPR (+SIFE, 5% kappa plasma cells in marrow)	351	Yes	No	Yes	Yes	N/a
29	CR (5% lambda plasma cells in marrow)	363	Yes	No	Yes	N/a	Yes

*PB* Peripheral blood, *BM* bone marrow, *CR* complete hematologic response, *VGPR* very good partial response, *PR* partial response, *SIFE* serum immunofixation electrophoresis, *UIFE* urine immunofixation electrophoresis.

Of the 13 patients with follow-up testing, ten had renal involvement and five had cardiac involvement at baseline. At time of MRD measurement, seven patients (70%) had a renal response. Two additional patients achieved a renal response at 1 month and 1 year later with no additional treatment. Renal response could not be assessed in the one patient with MRD negativity due to <500 mg/day of proteinuria at time of diagnosis. Of those with cardiac involvement, 3 (60%) had a cardiac response at the time of MRD assessment.

Although persistent disease can be detected with traditional measures, more sensitive techniques to assess MRD such as multiparametric flow cytometry (MFC), mass spectrometry, or NGS may be more informative. As demonstrated in multiple myeloma [7], detection of MRD may provide prognostic information, although test sensitivity should be considered. A minimum sensitivity of 1 × 10^−5^ is required based on multiple myeloma criteria, but a sensitivity threshold is not established in AL amyloidosis. MFC and next generation flow cytometry (NGF) have a sensitivity of 2.3 × 10^−6^ and 1 × 10^−5^, respectively [6, 11]. NGS, as used in this trial, has a sensitivity of 1 × 10^−6^. At this level of detection, an abnormal clone was detected in 86% of patients at baseline. A sensitivity of 97–100% was reported using other methods of MRD detection in AL amyloidosis [11–13].

The ability to detect MRD posttreatment is also important. In AL amyloidosis, MFC and NGF have detected MRD in 55–60% of patients with a hematologic CR [6, 11]. Matrix-assisted laser desorption/ionization-time-of-flight mass spectrometry detected residual disease in the serum of 12% of patients with a hematologic CR [14]. In our series only four patients achieved a hematologic CR, but MRD was detected in all four patients (100%) and overall in 92% of patients with a detectable clone pretreatment.

Achievement of MRD negativity may be of critical importance in AL amyloidosis, a disorder in which life-threatening organ dysfunction can worsen due to low-level toxic light chains. MRD negativity with MFC has been associated with improved progression free survival [15], as well as a trend toward improved organ function [6]. Despite this, it is important to note that many patients in our study achieved an organ response despite MRD positivity. The possibility of organ improvement in the presence of MRD must be noted in this population in whom the risk of treatment toxicity is high. It is possible that additional therapy aimed only at achieving MRD negativity may result in excess toxicity in already fragile patients. In patients with worsening organ function, MRD testing may guide additional therapy, but in those with continuing organ improvement, especially in the setting of poor treatment tolerance, close monitoring without treatment may be considered.

The limitations of this study include the small sample size and limited follow-up testing. Lack of uniformity in time to MRD specimen collection could hinder interpretation of organ responses, which often occur later. Despite these limitations, this study demonstrates the feasibility of using NGS to identify a clone and track MRD in AL amyloidosis.

MRD testing could have an important role in detecting persistence of a dangerous residual clone in AL amyloidosis and may provide evidence for additional treatment in patients with persistent or worsening organ dysfunction. Additional trials are needed to determine the most effective manner of assessing MRD and to evaluate the impact of MRD on patient outcomes and decision making. NGS is a sensitive method for detecting MRD and could be utilized in future studies.
